# Characteristics of Patients With Complex Limb Pain Evaluated Through an Interdisciplinary Approach Utilizing Magnetic Resonance Neurography

**DOI:** 10.3389/fpain.2021.689402

**Published:** 2021-05-31

**Authors:** Emily M. Johnson, Daehyun Yoon, Sandip Biswal, Catherine Curtin, Paige Fox, Thomas J. Wilson, Ian Carroll, Amelie Lutz, Vivianne L. Tawfik

**Affiliations:** ^1^Department of Anesthesiology, Perioperative and Pain Medicine, Stanford University School of Medicine, Stanford, CA, United States; ^2^Department of Radiology/Musculoskeletal Imaging, Stanford University School of Medicine, Stanford, CA, United States; ^3^Division of Plastic and Reconstructive Surgery, Department of Surgery, Stanford University School of Medicine, Stanford, CA, United States; ^4^Department of Neurosurgery, Stanford University School of Medicine, Stanford, CA, United States

**Keywords:** magnetic resonance neurography, pain, complex regional pain syndrome, neuropathy, interdisciplinary, neurolysis

## Abstract

Patients with persistent complex limb pain represent a substantial diagnostic challenge. Physical exam, and tests such as nerve conduction, are often normal even though the patient suffers from severe pain. In 2015, we initiated a team-based approach to evaluate such patients. The approach included physicians from several specialties (Anesthesiology/Pain Medicine, Radiology, Plastic Surgery, Neurosurgery) combined with the use of advanced imaging with Magnetic Resonance Neurography (MRN). This preliminary case series discusses MRN findings identified in patients with previously difficult-to-diagnose peripheral limb pain and describes how this combination of approaches influenced our diagnosis and treatment plans. We extracted demographics, patient characteristics, presenting features, diagnostic tests performed, treatments provided, referral diagnosis and the diagnosis after interdisciplinary team evaluation from patient charts. We evaluated MRN and electrodiagnostic studies (EDX) ability to identify injured nerves. We compared abnormal findings from these diagnostics to patient reported outcome after ultrasound-guided nerve block. A total of 58 patients, 17 males and 41 females, were identified. The majority of patients presented with lower extremity pain (75%) and had prior surgery (43%). The most commonly identified abnormality on MRN was nerve signal alteration on fluid sensitive sequences, followed by caliber change and impingement. Comparing the outcome of diagnostic nerve blocks with abnormal nerve findings on MRN or EDX, we found that MRN had a sensitivity of 67% and specificity of 100% while for EDX it was 45 and 0%, respectively. After interdisciplinary discussion and imaging review, a more specific diagnosis was produced in 78% of evaluated cases opening up additional treatment pathways such as nerve-targeted surgery, which was performed in 36% cases. This descriptive case series demonstrates that a majority of patients evaluated by our team for complex limb pain were women with lower extremity pain resulting from surgery. In addition, an interdisciplinary team evaluation and the use of the moderately sensitive but highly specific MRN imaging modality resulted in a change in diagnosis for a majority of patients with complex limb pain. Future studies investigating patient outcomes after diagnosis change are currently underway based on the findings of this preliminary study.

## Introduction

One third of adults in the United States currently suffer from chronic pain at a huge cost to society (1). Persistent limb pain after injury or surgery is common (2, 3). These patients present a unique diagnostic challenge as the initial trauma can include injury to multiple tissue types such as muscle, bone, and nerves (4). Identification of the underlying pain generator is further complicated by the fact that many patients have difficulty localizing the pain. Thus, clinicians face a challenging diagnostic process and can often only provide a non-specific diagnosis, which limits targeted therapeutic options. A clear diagnosis of the pain generator is required for targeted treatment of pain.

Challenges in diagnosis and treatment are not unique to the field of pain management. Other fields utilize interdisciplinary teams and advanced diagnostics to focus the diagnosis and guide the treatment plan in the care of complex patients (5). For example, a broad team of specialists evaluates children with complex craniofacial abnormalities to help guide treatment (6). Complex cancers are often evaluated by interdisciplinary tumor boards, which review the imaging and diagnostics and develop a targeted treatment plan. Such teams have been shown to improve the accuracy of pre-operative staging (7). According to the International Association for the Study of Pain (IASP), interdisciplinary treatment harnesses multimodal treatment by a multidisciplinary team working together to assess and ultimately treat patients, with shared goals in mind (8). Consistent with this approach we established an interdisciplinary team to evaluate patients with chronic pain where a clear pain generator had not been identified through the standard evaluation pathway. This team included clinicians from relevant specialties (Anesthesiology/Pain Medicine, Radiology, Plastic Surgery, Neurosurgery, Psychology, and Physical Therapy). Ultimately, we aim to facilitate interdisciplinary communication about diagnostically challenging patients with peripheral limb pain and improve diagnostic accuracy.

A key tool utilized by this interdisciplinary pain team was advanced magnetic resonance imaging (MRI) of the peripheral nerves. Magnetic resonance neurography (MRN) (9–11), a subfield of MRI dedicated to imaging peripheral nerves, can provide excellent anatomic delineation of peripheral nerves (12). It is a technically demanding approach, which requires careful optimization of imaging sequence parameters but has been shown in limited studies to refine diagnosis (13). To determine the sensitivity and specificity of MRN, and the more commonly used electrodiagnostic studies (EDX), we compared abnormal findings from these studies to the outcomes patients reported after ultrasound-guided nerve block, the most robust technique to localize a painful nerve.

In this preliminary case series, we report on 58 patients who were treated by our interdisciplinary pain team during a 3-year period. Our aim was to share our experience with this novel team approach, discuss MRN findings identified in patients with previously difficult-to-diagnose peripheral limb pain and describe how this combination of approaches influenced our diagnosis and treatment plans.

## Materials and Methods

We conducted a single-center retrospective case series to understand the impact of an interdisciplinary team approach on the diagnosis and treatment of patients with refractory limb pain. All data were collected in accordance with the ethical standards of the Institutional Review Board at Stanford University School of Medicine. Extraction of data from patient charts was approved as a chart review protocol; consequently, no patient consent was required for inclusion in the study.

### Patients

All patients included in the study had upper or lower extremity limb pain and were referred to Stanford Hospital & Clinics Pain Management Clinic for evaluation between January 1, 2015 and November 1, 2018. Patients were included if they received evaluation by MRN and were discussed at our bimonthly, interdisciplinary care team meeting (see below).

### Interdisciplinary Nerve Team

The interdisciplinary team was established in 2015 to discuss complex cases where a diagnosis was not clear using standard approaches (physical exam, neurological exam, x-rays, standard MRI etc.). The team consisted of anesthesiologists subspecialized in Pain Medicine (IC, VT), peripheral nerve surgeons collaborating from plastic surgery and neurosurgery (CC, PF, TW), musculoskeletal radiologists (SB, AL) and an MRI physicist (DY). We also benefited from the expertise of physical therapists and pain psychologists from the Stanford Pain Medicine Clinic who attended meetings when able, but also independently evaluated a majority of our patients (see section Results). During the team meeting, the pain medicine specialist or surgeon would describe the history focusing on the relevant pain description, current and past medications, and pertinent physical exam such as distribution of sensory and motor changes. Results from other diagnostics such as electromyography/nerve conduction studies (referred to as electrodiagnostic studies, EDX), ultrasound-guided blocks, and any other treatments were reviewed. Next, the radiologist would review and describe the results of MRN. This was followed by a discussion of the patient's case to generate a consensus diagnosis and multimodal treatment plan. Following the meeting, any subsequent diagnostic tests were ordered and the agreed upon treatment plan was communicated to the patient.

### MRI of Peripheral Nerves (Magnetic Resonance Neurography)

Multiple MR sequences of different imaging planes and contrast were employed for evaluating peripheral nerves. Patients were scanned in the Discovery MR 750 3T MRI scanners (GE Healthcare, Waukesha, WI, U.S.A.). The adopted MR pulse sequences and their sequence parameters are briefly summarized in Supplementary Table 1. T2-weighted sequences with fat-saturation were used for detecting abnormal signal changes on nerves whereas T1-weighted sequences were mostly used for identifying dominant anatomic markers (muscles, bone, and fat) and secondary abnormalities such as fatty infiltration or scarring. The short-tau inversion recovery (STIR) sequence was used optionally to obtain a robust reference for water-only images in cases of fat-saturation failure for the T2-weighted sequences. Images were protocoled and read by our expert radiologists (SB, AL) and parameters optimized by our MR physicist (DY).

### Data Collection

Medical records were reviewed retrospectively. Information collected included: demographics (age, sex, race), age at symptom onset, duration of symptoms, limb affected, reported initiating event, referral diagnosis (diagnosis upon starting care at Stanford Health Care), diagnosis after team evaluation, diagnostics performed [nerve blocks, EDX, standard MRI, nuclear medicine scans, positron emission tomography/magnetic resonance imaging (PET/MRI) research study], and treatments initiated (medication recommendations, physical therapy, pain psychology, intravenous infusions, radiofrequency or cryo-ablation, botox injection, peripheral or spinal cord stimulation, nerve-targeted surgery).

### Data Analysis

Demographic data are presented as mean ± SD or percentages, as indicated in the results and tables. Nerve abnormalities identified by MRN review were classified into the following types: signal alteration, caliber change, impingement/focal deviation/fat obliteration, mass or mass-like lesion, and direct trauma/disruption.

Nerve abnormalities between MRN and EDX were compared. Out of the 26 patients who had EDX and MRN, there were 3 different scenarios we observed: (1) Patients came with EDX already performed and we added MRN for additional diagnostic information (6/26 patients), (2) We ordered MRN and EDX simultaneously (15/26 patients), (3) We felt we needed more diagnostic information after discussion of MRN findings and therefore ordered EDX (5/26 patients). The sensitivity and specificity of MRN and EDX in detecting nerve involvement in the pain syndrome was determined using the outcome of a diagnostic nerve block as the gold standard. This is based on the fact that local anesthetic blockade inhibits all conduction of a given peripheral nerve from reaching the central nervous system and results in regional anesthesia limited to the dermatome innervated by that nerve. Consequently, an improvement in pain with nerve block would suggest at least some contribution of that nerve to the patients' pain condition. For calculation of sensitivity and specificity, if a patient reported decreased pain after local anesthesia block of a detected abnormal nerve this was classified as a true positive. If a patient reported no change in pain after local block of a nerve without detected abnormalities this was classified as a true negative.


$$\begin{array}{l} \text{ Sensitivity }=\;\frac{\text{Abnormal nerve on MRN AND nerve block resulting in decreased pain}}{\text{All nerve blocks resulting in decreased pain}}\\ \text{ Specificity }=\;\frac{\text{No abnormal nerve on MRN AND nerve block resulting in no change or increasedpain}}{\text{All nerve blocks resulting in no change or increased pain}} \end{array}$$

Furthermore, the sensitivity of MRI was compared to that of EDX using the one-sided McNemar's test with significance of 5% and the odds ratio. To evaluate the impact of our team approach on diagnosis, the referral diagnosis was compared with the diagnosis after our interdisciplinary team meeting including discussion of MRN results.

## Results

### Patient Data

A total of 17 male and 41 female patients met our inclusion criteria. The interdisciplinary pain team evaluated these patients between January 1, 2015 and November 1, 2018 (Table 1). Patients had a mean age of 51 (±SD 16) years and mean duration of symptoms of 9 (±SD 16) years. Patients were predominantly Caucasian (76%). Three-quarters of patients presented with either unilateral (66%) or bilateral (9%) lower extremity pain, compared to only 25% of patients with pain in either one or both upper extremities (Table 2). None of the 58 patients included were amputees. Most patients were able to attribute their pain to one or more initiating events such as surgery (43%), trauma (without fracture or diagnosed injury, 22%), or fracture (12%). Twenty-four percent of patients reported no clear triggering event (Table 2).

**Table 1:** Demographics and patient characteristics.

Number of participants	58
Male	17 (29%)
Female	41 (72%)
Age current (years)	51 ± 16
Age at symptom onset (years)	44 ± 16
Female	42 ± 16
Male	48 ±16
Duration of symptoms (years)	9 ± 16
Race	
White	44 (76%)
Asian	3 (5%)
Black or African American	1 (2%)
Native Hawaiian or Pacific Islander	1 (2%)
American Indian or Alaska Native	1 (2%)
Other	5 (9%)
Unknown	4 (7%)

*Numbers are reported as n (%) or average ± standard deviation*.

**Table 2:** Presenting features and inciting event.

**Limb affected**	% patients
Upper (unilateral)	13 (22%)
Lower (unilateral)	38 (66%)
Upper (bilateral)	2 (3%)
Lower (bilateral)	5 (9%)
Initiating event^*^	% patients
Surgery	25 (43%)
Trauma (no fracture or diagnosed injury)	13 (22%)
Fracture	7 (12%)
Sprain	3 (5%)
Other	5 (9%)
Unknown	14 (24%)

*Numbers are reported as n (%)*.

* *Several patients listed both “fracture and surgery” or “trauma and surgery” as their inciting event and therefore totals do not add up to 100%*.

### Diagnostics Performed

After initial evaluation, all patients (100%, Table 3) underwent MRN as a diagnostic imaging exam to evaluate potential nerve involvement, since it was a requirement for study inclusion. Additional diagnostics performed included nerve block (81%), EDX (45%), standard MRI (41%), nuclear medicine bone scan (2%), and enrollment in an ongoing PET/MR imaging research study (26%; ClinicalTrials.gov, NCT03556137).

**Table 3:** Diagnostics performed.

**Diagnostic test**	**% patients**
MR neurography	58 (100%)
Nerve block	47 (81%)
EDX	26 (45%)
Standard MRI	24 (41%)
PET/MR study	15 (26%)
NMR bone scan	1 (2%)

*Numbers are reported as n (%)*.

### Nerve Abnormalities on MRN

**Figure 1:**
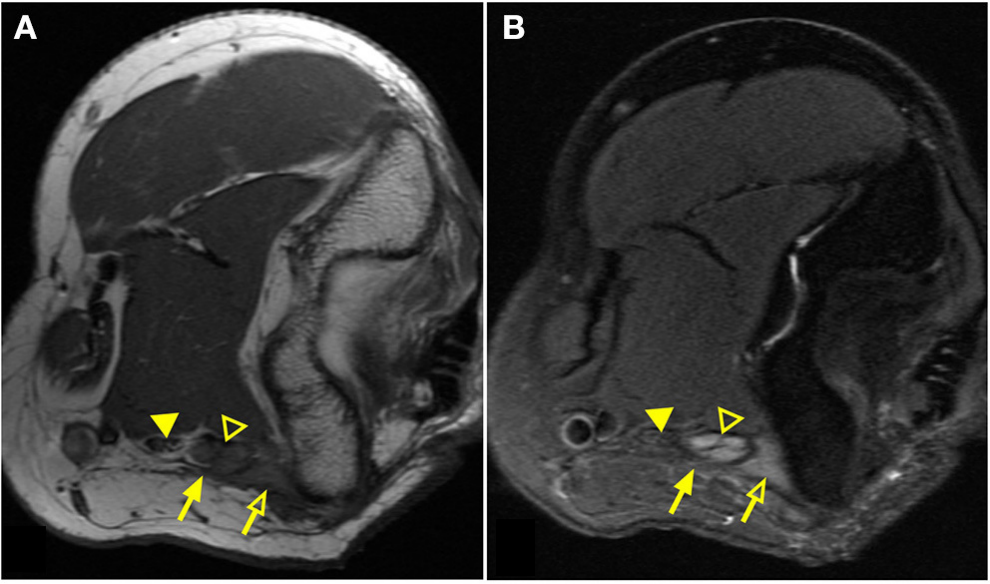
Magnetic resonance neurography (MRN) of the elbow demonstrates clear nerve abnormality. Elbow images of a patient with persistent/increasing ulnar nerve distribution symptoms after ulnar nerve transposition surgery. T1-weighted image **(A)** demonstrates scar tissue/fibrosis (solid arrow) around the ulnar nerve (open arrowhead) in greater detail. T2-weighted image **(B)** presents significantly increased signal of the ulnar nerve (open arrowhead) in comparison to the normal median nerve (solid arrowhead). Edematous muscle (open arrow) is clearly distinguished with increased signal on the T2-weighted image.

MRN scans revealed nerve abnormalities in 71% of patients including signal alteration (60%), caliber change (26%), impingement (22%), mass or mass-like lesion (7%), and direct trauma/disruption (2%) in nerves that were consistent with the patients presenting regional symptoms (Table 4). Importantly, 38% of patients met criteria for more than one category of radiologic findings and only 29% had no noted nerve abnormality. Figure 1 demonstrates striking signal alterations of nerves and surrounding tissues visualized with MRN in one patient included in this study. In this patient, MRN at the level of the elbow shows scar/fibrosis on the T1-weighted image (Figure 1A) around the ulnar nerve with clear signal alteration and caliber change and edematous muscle on the T2-weighted image (Figure 1B).

**Table 4:** MR neurography findings in all patients and in the subset who underwent surgery as a treatment option.

Radiologic findings^*^	**% patients**	**% patients who underwent surgery as a treatment**
Signal alteration	35 (60%)	12 (57%)
Caliber change	15 (26%)	8 (38%)
Impingement/focal deviation/fat obliteration	13 (22%)	3 (14%)
Mass or mass-like lesion	4 (7%)	3 (14%)
Trauma/disruption	1 (2%)	1 (5%)
None	17 (29%)	5 (24%)
>1 finding	22 (38%)	8 (38%)

* *Note that there were 58 patients total and 21 patients who underwent surgery, however, a portion of patients met criteria for more than one radiologic finding and therefore totals do not add up to 100%*.

*Numbers are reported as n (%)*.

Since 43% of patients identified surgery as the initiating event for their pain condition, implying that surgery itself could trigger complex limb pain, we were particularly interested in understanding the MRN findings of the subgroup of patients who underwent Nerve Team recommended surgery as a treatment for their pain (see below). Of the patients who underwent surgery (Table 4), MRN findings were positive in 76% and included: signal alteration (57%), caliber change (38%), impingement (14%), mass or mass-like lesion (14%) and direct trauma/disruption (5%) with 38% meeting criteria for more than one category of radiologic abnormalities. Caliber change was seen more often in patients who underwent surgery (38%) compared to those patients who did not (26%).

### MRN vs. EDX

Twenty-six patients underwent EDX in addition to MRN, however, there was agreement on the exact abnormal nerve(s) between MRN and EDX in only 6 patients (Table 5). In 18 of 26 cases (69%), EDX did not correlate with the abnormal nerve identified by MRN. In 10 of 26 cases (38%) MRN did not correlate with the abnormal nerve identified by EDX. Nerve block outcome was available for 47 patients who received MRN and 23 patients who received EDX and MRN (since all patients had MRN for study inclusion). The sensitivity/specificity of MRN was 67% (28 of 42)/100% (5 of 5) as shown in Table 6 and for EDX was 45% (10 of 22)/0% (0 of 1) as shown in Table 7. Table 8 presents a 2 × 2 contingency table comparing the result of MRN and EDX for the 23 patients who underwent both MRN, EDX, and had a positive nerve block outcome. The *p*-value from one-sided McNemar's test was 0.07 and the odds ratio was 3.0.

**Table 5:** Frequency of different types of findings between MRN and EDX.

**Intervention**	**Number of patients**
Abnormal nerves found by EDX are a subset of abnormal nerves found by MRN	10
Abnormal nerves found by MRN are a subset of abnormal nerves found by EDX	2
EDX and MRN identified the exact same set of abnormal nerves	6
None of above	8

*In general, MRN found more nerves with abnormalities than EDX in the symptomatic area (n = 26)*.

**Table 6:** The outcome of nerve blocks in the area examined by MRN.

	**Outcome of nerve block**	
**MRN findings**	**Decreased pain**	**No change or worsening of pain**
Abnormal nerve on MRN	28	0
No abnormal nerve on MRN	14	5

*The sensitivity and specificity were 67% (28/42) and 100% (5/5). n = 47*.

**Table 7:** The outcome of nerve blocks in the area examined by EDX.

	**Outcome of nerve block**	
**EDX findings**	**Decreased pain**	**No change or worsening of pain**
Abnormal nerve on EDX	10	1
No abnormal nerve on EDX	12	0

*The sensitivity and specificity were 45% (10/22) and 0% (0/1). n = 23*.

**Table 8:** The detection accuracy for the causative nerve damage between MRN and EDX based on the positive outcome of nerve blocks.

		**EDX**	
		**Detection success**	**Detection failure**
MRN	Detection success	7	9
	Detection failure	3	4

*The odds ratio was 3.0 (9/3), suggesting a higher sensitivity for MRN than for EDX. n = 23*.

### Diagnosis After Team Evaluation

Overall, the referral diagnoses before interdisciplinary team evaluation tended to be less specific such as: complex regional pain syndrome not otherwise specified (NOS), neuropathy without specified nerve involved, and regional diagnoses such as “limb pain” or “joint pain.” Ultimately, we changed the diagnosis in 45 out of 58 patients, 78% of cases (Table 9). In the majority of these cases (65.5% of total) our evaluation resulted in a more precise diagnosis [e.g., a specified neuropathy or Complex Regional Pain Syndrome (CRPS) Type II with specified nerve involvement].

**Table 9:** Comparison between referral diagnosis and diagnosis after nerve team evaluation.

		**Diagnosis after interdisciplinary nerve team evaluation**					
		**CRPS I**	**CRPS II**	**CRPS NOS**	Neuropathy^*^	**Joint dysfunction**	**Total**
**Referral diagnosis**	CRPS I	**3**	6	0	1	0	10
	CRPS II	1	**1**	0	0	0	2
	CRPS NOS	2	4	**0**	0	0	6
	Neuropathy^*^	1	0	0	**9**	0	10
	Neuropathy NOS	0	0	0	2	0	2
	Limb pain	0	2	0	5	1	8
	Joint pain	0	5	0	7	0	12
	Pain NOS	0	1	0	5	2	8
	Total	7	19	0	29	3	58

* *Neuropathy of a specified peripheral nerve. NOS, not otherwise specified*.

*Values in bold and highlighted represent the diagnoses that did not change after interdisciplinary nerve team evaluation*.

### Treatments Offered

Multimodal treatment plans were instituted in all patients (Table 10). Medications were changed in 95% of patients and 78% of patients were referred to physical or occupational therapy and pain psychology for one-time evaluations or ongoing therapy. Intravenous infusions (i.e., ketamine or lidocaine) with an inpatient or outpatient protocol were implemented in 40% of cases. Importantly, targeted treatments directed at the peripheral nerves we imaged were performed as follows: pulsed radiofrequency neuromodulation (12%), botox injection (12%), cryoablation (10%), dorsal column/dorsal root ganglion spinal cord stimulator (10%), or peripheral nerve stimulator (9%). Targeted peripheral nerve surgery was performed in 36% of cases, with 33% of those patients noting that a prior surgery triggered their complex limb pain. Before the corrective surgery recommended by the Nerve Team, 100% of patients had chart documented improvement in pain with an ultrasound-guided nerve block. Most frequently, surgical decompression and untethering (neurolysis) was performed (90% of surgeries).

**Table 10:** Treatment and management.

**Intervention**	**% patients**
Medication changes	55 (95%)
Physical/occupational therapy	45 (78%)
Pain psychology	45 (78%)
Intravenous infusion (e.g., Ketamine)	23 (40%)
Surgery	21 (36%)
Pulsed Radiofrequency neuromodulation	7 (12%)
Botox injection	7 (12%)
Cryoablation	6 (10%)
Spinal cord stimulator	6 (10%)
Peripheral nerve stimulator	5 (9%)

*Numbers are reported as n (%)*.

## Discussion

Our results suggest that an interdisciplinary team evaluation combined with the moderately sensitive, but highly specific, MRN imaging modality can change diagnosis for a majority of complex patients with limb pain. Future studies focused on outcomes after diagnosis change would further strengthen our findings and are currently underway at our institution.

In our results, MRN identified more abnormal nerves than EDX, which led to higher sensitivity to detect nerve damage that correlated with pain. The higher sensitivity of MRN may stem from the improved spatial localization of small nerves. EDX measurements are acquired through local volume conduction of electric signal between manually placed electrode and target nerves (14). This process is susceptible to human errors, individual anatomic variation, and bias for large nerves (15). EDX can also be a painful test and hard for a patient in pain to tolerate. The findings for MRN were extracted from MRN reads conducted in our hospital by our faculty radiologists (SB and AL). Our current clinical practice for MRN review is based on a qualitative description of abnormalities and semi-quantitative scoring (absent, mild, moderate, severe), but not true quantitative scores. While it would be ideal to perform a receiver operating characteristic (ROC) analysis for comparing the diagnostic accuracy between MRN and EDX, this would not be possible given the available data from current clinical radiology reports. The adoption of more quantitative techniques like MRI relaxometry or diffusion MRI in the MRN protocol may enable this type of ROC analysis which requires a binary input but would require a separate research study to confirm validity as it is not routinely performed in clinical practice. That said, the outcome of McNemar's test did not show a statistically significant difference between the sensitivities of MRN and EDX (*p* = 0.07). Moreover, the odds ratio of 3.0 supports the potential for MRN to improve sensitivity in diagnosis, however, the magnitude of this effect requires further study.

It has been previously described that MRN in isolation is subject to both false positives and false negatives (12). One approach to improve diagnostic specificity is to utilize an interdisciplinary team to present the history and physical examination alongside radiology expertise to review the MRN thus allowing for more precise diagnosis and increased collaboration (16). MRN must be interpreted in the context of the history and physical examination findings to minimize false positives. While our team approach provided this context, we were nevertheless surprised by the complete lack of false positives on MRN in our study. The limited sample size may have additionally contributed to the much higher specificity of MRN compared to EDX. The specificity of EDX can be quite high in conditions such as polyneuropathy and radiculopathy in which multiple nerves or the nerve roots themselves are implicated (17) and given the focal nature of the abnormalities detected in our study, it is possible that they did not result in conduction abnormalities.

Our retrospective analysis demonstrated that team management combined with MRN refined diagnosis in 78% of cases. We are not the first to report that MRN can solidify diagnosis in complex cases of neuropathic limb pain. Chhabra et al. (13), demonstrated that MRN can change surgical planning by increasing surgeon confidence in approach and surgical success. General, non-specific diagnoses are often treated with general, systemic treatments, whereas specific diagnoses can result in a more tailored, targeted approach. In our study, such specific approaches included radiofrequency ablation, cryoablation, botox injection, peripheral nerve stimulator (PNS) placement, and surgical neurolysis. The overall low prevalence of PNS placement (9%) is likely due to the timeframe analyzed (2015–2018) for this case series. Since 2018, the use of PNS to target nerves involved in diverse pain conditions has increased substantially as the use of ultrasound has become widespread and newer percutaneous devices are easily placed under local anesthesia (18). The advances in this area continue to impact the availability of specific treatment options for our patients.

One unique aspect of limb pain is the diagnosis of CRPS (19). CRPS is a neuropathic pain syndrome and has two types: CRPS Type I (no known nerve injury) and CRPS Type II (known injury leading to pain) (20). If a nerve is identified that contributes to the underlying pathology, targeted treatment directed at the injured nerve can be offered. Prior to team evaluation, CRPS Type I was more prevalent than CRPS Type II (89 vs. 11%) in our cohort. After interdisciplinary evaluation with MRN, CRPS Type I was less prevalent than CRPS Type II (27 vs. 73%), which is contrary to published literature (19). This suggests that some, if not most, CRPS I may actually be CRPS II with an occult nerve injury. With better imaging techniques guided by specialized expertise in examination of the peripheral nervous system, we believe more of these occult nerve injuries will be identified and CRPS II could become a more prevalent diagnosis. This is more than just semantics and terminology, since identification of the problematic nerve in CRPS II opens up additional targeted treatment options as illustrated in our patients. Treatment for CRPS is time sensitive with early treatment producing better outcomes for patients (21). This makes timely and accurate diagnosis critically important and necessary (22). Importantly, CRPS is a pain condition often triggered by minor surgery (23) and the risk of CRPS recurrence after repeat surgery on the CRPS affected limb is estimated to be about 13% making such a treatment option controversial (24). Since 43% of our patients, many of whom carried a diagnosis of CRPS, ascribed their poor outcome (i.e., persistent limb pain) to previous surgery, recommending surgery as a treatment option was a complicated shared decision with each patient. In the 7 cases in whom surgery was the identified trigger and who underwent subsequent Nerve Team recommended surgery as a treatment, all had lower extremity pain and incomplete prior nerve releases, or nerves found to be associated with scar tissue/fibrosis. Together our results outline a role for surgery as a treatment for complex limb pain, even in those patients with a history of surgery-related pain and/or CRPS.

There are several limitations to the current study. We have shown that our approach frequently results in a change in diagnosis, however, whether this improves outcomes remains an open question for further study. Given the retrospective nature of this work and the diversity of patients we cared for over a 3-year time period, finding consistent, reportable outcomes that could then be correlated back to the change in diagnosis was not possible. Additionally, such correlations would be subject to many confounding variables that we did not feel it would be useful. Instead, based on the current findings, we are designing a prospective study to evaluate whether interdisciplinary team management with MRN changes patient-reported outcome measures which will be an important next step to clarify the benefit of our approach to patients (25, 26). Additionally, as a retrospective, single center, chart review, case series, there are several biases and limitations inherent to such a study design. Ideally, we would like to compare patients evaluated by MRN and team meeting discussion to a group that had interdisciplinary team discussion alone. Given the goals of the bimonthly team meeting to review MRN findings in the context of patient history, and the substantial time commitment required to discuss the patients presented, we were unable to achieve such a comparison group. Additionally, given the low numbers of patients who underwent MRN and/or EDX as well as ultrasound-guided nerve block, and the lack of vehicle (placebo) nerve block injection, our estimates for sensitivity and specificity for these diagnostics must be taken in the context of these limitations. Finally, we recognize that widespread adoption of the practices we propose here is challenging since the interdisciplinary team model involves many physicians from varied specialties, coordination of schedules, and advanced imaging.

Combining novel imaging techniques with interdisciplinary team meetings is an important approach to help care for patients with refractory, difficult-to-diagnose pain syndromes. Such an approach may help uncover the underlying etiology of complex pain cases and contribute to new and more directed management plans. We hypothesize that such targeted and mechanism-specific treatments will ultimately result in better outcomes which we are now positioned to evaluate.

## Data Availability Statement

The raw data supporting the conclusions of this article will be made available by the authors, without undue reservation.

## Ethics Statement

The studies involving human participants were reviewed and approved by Institutional Review Board at Stanford University School of Medicine. Written informed consent for participation was not required for this study in accordance with the national legislation and the institutional requirements.

## Author Contributions

EJ, DY, and VT performed chart review and wrote the manuscript. EJ, DY, AL, and VT performed analyses. SB and AL read all the imaging studies. All authors contributed to patient care, case review, manuscript editing, and approved the final submission.

## Conflict of Interest

DY, SB, and AL have received research support from GE Healthcare. The remaining authors declare that the research was conducted in the absence of any commercial or financial relationships that could be construed as a potential conflict of interest.
