# Supplementary material for: Characteristics of Patients With Complex Limb Pain Evaluated Through an Interdisciplinary Approach Utilizing Magnetic Resonance Neurography
Source: Front Pain Res (Lausanne). 2021 May 31;2:689402. doi: 10.3389/fpain.2021.689402 (PMC8915577; doi:10.3389/fpain.2021.689402)
Supplement: Supplementary file 1 [file Table_1.DOCX]

|  | | **2D Axial T1** | **2D Axial T2 with fat saturation** | **2D Sagittal with short-tau inversion recovery** | **2D Coronal T2 with fat saturation** | **2D Coronal T1** |
| --- | --- | --- | --- | --- | --- | --- |
| **Parameters** | **Echo Time** (ms) | 20 | 54 | 54 | 54 | 12 |
|  | **Repetition Time** (ms) | 898 | 5131 | 5987 | 3844 | 669 |
|  | **Resolution** (mm) | 0.77 x 1.43 x 4 | 0.83 x 1.43 x 4 | 1 x 1.43 x 5 | 0.77 x 1.43 x 4 | 0.77 x 1.43 x 4 |
|  | **Bandwidth** (KHz) | ±41.67 | ±31.25 | ±31.25 | ±31.25 | ±31.25 |
|  | **Flip Angle** (°) | 111 | 111 | 111 | 111 | 111 |
|  | **Echo Train Length** | 4 | 15 | 16 | 14 | 3 |
|  | **Number of Excitation** | 2 | 3 | 2 | 3 | 3 |

**Supplemental Table 1.** Adopted MR imaging sequences and their parameters. All sequences are fast-spin-echo (FSE) sequences. The dominant contrast of the FSE sequences are presented in their names (T1 or T2).
